# Alternative metrics for measuring the quality of articles and journals

**DOI:** 10.3332/ecancer.2013.ed18

**Published:** 2013-04-08

**Authors:** Richard Smith

**Affiliations:** Director, UnitedHealth Chronic Disease Initiative; Chair, Patients Know Best

The traditional measure of the quality of scientific articles and journals is the impact factor, but there are many problems with impact factors [1]. What are the other ways in which articles and journals might be measured?

I’ve fretted about this problem since 1991 when I became the editor of the BMJ and chief executive of the BMJ Publishing Group responsible for about another 15 journals. In those days academics in Britain and editors of specialist journals were not familiar with impact factors, but once they began to be used to measuring the performance of academic departments—and, most important of all, allocating money to them—then academics began to be obsessed by impact factors. Editors too became obsessed and would weep when their journal impact factors fell by one decimal point.

The impact factor of a journal for, say, 2012 (and it is measured annually) is citations in 2012 to articles published in 2010-2011 divided by number of “citable” articles published in 2010-2011. The New England Journal of Medicine had an impact factor in 2012 of 53.298 (including three decimal points gives a spurious sense of precision and importance) but most journals have impact factors under 1. When it comes to journals, the impact factor has some validity because the articles in the journal being highly cited must be some sort of measure of impact. Citations do, however, come mostly from researchers, and so a “researcher to researcher” journal will have many more citations than a “researcher to clinician” or a “clinician to clinician” journal. So a journal that wants to appeal primarily to practising clinicians, most of whom are not researchers, may lose readers and usefulness as its impact factor rises.

It is, however, wholly unscientific to use the impact factor of a journal to measure the impact of a paper published within the journal—for the simple reason that the impact factor of journals is driven by a few articles being highly cited [1]. There is thus little correlation between the impact factor of a journal and the number of citations to individual articles published within it. This lack of correlation is one of the factors driving a search for alternative ways of measuring the quality of individual articles. The Public Library of Science (where I was on the board from 2004 to 2011) has led the way, and if you click on any article published by PLoS you will see at the top of the page something entitled “Metrics.” Let’s use as an example the most highly cited paper from PLoS Medicine, which ironically is entitled “Why most published research findings are false” [2]. It’s by the brilliant and iconoclastic researcher John Ioannidis.

When you click on Metrics what hits you first is a graph showing accumulated page views and PDF downloads of the article. An article where the graph continues upwards is probably more important than one where there is a brief ascent and then a levelling off. And in contrast to impact factors, which are published only once a year and tell you about what was published rather than what is being published, page views are shown in real time. Ioannidis’ article had had on 13 March 2013 611 448 page views (516 433 through PLoS and 95 015 through PubMed Central), and the graph far from flattening off seems to be growing faster.

There are other data on usage, but the next section shows citations—not from one database as is the case with impact factors but from Scopus (915 citations), Crossref (481), PubMed Central (178), ISI Web of Science (859), which is used for impact factors, and Google Scholar (1401). These too are in something close to real time and avoid the criticism that to use one database, as does the impact factor, is too narrow. The next section gives data from social networks. Academics may have been snooty about social networks, but many academics are now recognising their importance in the modern world and are following major organisations and media in using them extensively. The number of your Twitter followers is rapidly becoming the prime measure of your worth, much more so than the number of degrees or honours you have. PLoS gives data for CiteUlike (360), Connotea (18), Facebook (9612), and Twitter (477). One of the defects of impact factors is that they measure impact in only one world, the research world, and increasingly funders of research are interested in not just the scientific impact of the research they fund but also the social impact. Has the research led to health, economic, or social benefit? In Britain the Higher Education Funding Council has ignored resistance from basic scientists and will measure social impact in its next assessment of research performance of academic departments. It will do this not through metrics but through case studies, which might include some metrics like numbers of patents, quotes in the media, or inclusions in clinical guidelines. PLoS gives data for Trackbacks (3), Research blogging (9) Wikipedia (80), and Google. Finally, PLoS shows the number of comments made on the article (30).

The reviewer of this editorial criticised this piece as being too PLoS-centric, which is a fair criticism. He or she pointed me to ResearchGate, which seems to be primarily for scientists to get a reputation score. So this is something like the H-index, a way of measuring the reputation/ impact/influence of an individual scientist. I tried to sign up for ResearchGate but failed, partly because I don’t belong to an academic institution. Academia.edu is another website that provides analytics for individual scientists. I have concentrated on PLoS, but the broad point is that there are many groups trying to find better ways of measuring the impact of journals, articles, and scientists. We will probably go through a phase of “creative destruction” in which many of these innovations will disappear and a few survive, flourish, and become dominant.

Other publishers are beginning to introduce article level metrics, but most are going slowly and none have such a comprehensive set as PLoS. So the sad reality for now is that judgements on the quality of papers continue to be made on the basis of the journals in which they are published. It’s just too much work to read individual articles to judge their quality, and article level metrics are either not available or not used. This will surely change—but slowly unless organisations distributing money and honours pay much more attention to them. As I’ve said, journals can be more legitimately measured using impact factors, but there are potentially many other metrics that can be considered—for example, numbers of readers, revenue, profit, and geographical reach. When I was at the BMJ Publishing Group, we wanted our journals to be influential. But what is influence and how can it be measured? Our business staff were somewhat scornful of influence, arguing that it was too vague and that we should simply concentrate on revenue, profit, and the impact factor. This is the view of most publishers, but I and several of my colleagues weren’t interested in publishing journals that simply made lots of money and were often cited by researchers but had little influence in the broader world.

So I tried to come up with an operational way of defining and ultimately measuring influence. I saw six levels of influence. The top level (level one) is a change in the real world because of what a journal has published—for example, doctors change the way they treat patients or government policies change. Level two is setting an agenda or legitimising an issue—in the way, for example, the Lancet has influenced debates on human rights and health. Level three is leading by example and being followed, the way, for example, that PLoS has established article level metrics or how the BMJ introduced rapid responses. Level four is being quoted—in other journals, the mass media, parliament, or many other places. Citations are an example of level four, and metrics can be attached to level four more easily than to the higher levels. Level five is being paid attention to-for example, website hits or readership figures. Again metrics can be attached. Level six, the lowest level, is simply being known about, and clearly Barack Obama knowing about your journal is a more important measure of influence than your mother knowing about it. I went further and attempted to allocate scores and weightings to the different level, but my system has never been adopted.

The brutal truth is that it’s easy to measure and pay attention to impact factors but hard to develop new metrics and have them widely accepted. The appearance of article level metrics, the many creative attempts to find new metrics, the attempts by funding bodies to measure social impact are, however, signals of change.
